# 

**Published:** 1856-10

**Authors:** 


*Bromine as a specific in Pseudo-Membraneous Affections.*

M. Ozanam, in a paper presented to the Imperial Academy of Sciences, on the 26th of May, announces that bromine is a specific in the pseudo-membranous affections. He has treated successfully fourteen cases, two of which were cases of true croup. He employed either bromine or bromide of potassium. The dose was from one to ten grains a day.—*Med. Times and Gazette*, June.

